# Relationship between seminal plasma zinc and semen quality in a subfertile population

**DOI:** 10.4103/0974-1208.74153

**Published:** 2010

**Authors:** DMAB Dissanayake, PS Wijesinghe, WD Ratnasooriya, S Wimalasena

**Affiliations:** Department of Obstetrics and Gynaecology, University of Kelaniya, Sri Lanka; 1 Department of Zoology, University of Colombo, Sri Lanka; 2 Department of Chemistry, University of Kelaniya, Sri Lanka

**Keywords:** Semen parameters, subfertility, zinc

## Abstract

**RATIONALE:**

Current knowledge on the relationship between seminal zinc levels and different parameters of human semen is inconsistent.

**OBJECTIVES:**

To assess the relationship between seminal plasma zinc and semen quality using two markers; zinc concentration (Zn-C) and total zinc per ejaculate (Zn-T).

**DESIGN:**

The study was carried out as a cross-sectional study.

**SUBJECTS AND METHODS:**

Semen parameters of 152 healthy men undergoing evaluation for subfertility were assessed. Seminal plasma zinc levels were determined using flame atomic absorption spectrometry. Zn-C, expressed as *μ*g/mL, was multiplied by ejaculated volume to calculate Zn-T. Mann Whitney U test and Chi-square test were used to compare the zinc levels between different seminal groups when appropriate. Correlations were observed with Pearson’s correlation of coefficient. Analysis was carried out using SPSS 10.0 for windows software.

**RESULTS:**

Zn-C was low in 23 (15%) samples, while in 32 (21%) of the samples Zn-T was abnormal. The number of subnormal samples was high in the low-zinc groups compared with the normal-zinc groups, 15 vs. 8 (*P* > 0.05) for Zn-C and 28 vs. 4 (*P* < 0.001) for Zn-T. Zn-C was significantly high in the asthenozoospermics compared with the normal motile group; 138.11 *μ*g/mL (83.92) vs. 110.69 11 *μ*g/mL (54.59) (*P* < 0.05). Zn-T was significantly low in samples with hyperviscosity compared with samples with normal viscosity; 220.06 *μ*g (144.09) vs. 336.34 *μ*g (236.33) (*P* < 0.05). Conversely, Zn-T was high in samples with low viability compared with those with normal viability; 437.67 *μ*g (283.88) vs. 305.15 *μ*g (221.19) (*P* < 0.05). Weak correlations were found between Zn and some semen parameters. However, the correlation was negative between pH and Zn-C (r = –0.193, *P* < 0.05) as well as Zn-T (r = –0.280, *P* < 0.01). On the other hand, correlations were positive between Zn-T and sperm count (r = 0.211, *P* < 0.05).

**CONCLUSION:**

Count, motility, viability, pH and viscosity are affected by variations of seminal plasma zinc. Seminal plasma Zn-T is the better marker for assessing the relationship between zinc and semen quality.

## INTRODUCTION

The adult human body contains 1–3 g of zinc, about 0.1% of which is replenished daily.[1] Excessively high amounts of zinc has been reported in human seminal plasma by many authors, the mean ranges from 78.9 to 274.6 mg/L.[2–8] The major contributor of seminal plasma zinc is the prostate gland.[9,10] This high concentration of prostatic zinc ions comes into contact with sperm after their functional maturation. On the other hand, the seminal zinc ions get diluted by the vaginal and cervical fluids immediately after ejaculation. Therefore, it is not clear as to how this high zinc level in seminal plasma affects the sperm function. However, its protective role as an antioxidant and as an antibacterial agent is well documented.[7,11] It also provides a protective role against heavy metal accumulation (Pb) mediated by competition between them or due to the reduction of available binding sites.[12]

Although some beneficial effects of zinc on semen have been accepted, controversies continue regarding zinc levels between different subfertile groups as well as the relationship between zinc and semen parameters. Some authors reported significantly different seminal zinc levels between fertile and subfertile groups, indicating low seminal zinc levels in the subfertile populations,[2,13] while some others have shown that there is no difference between the two groups.[5,6,14]

Attempts have been made over the years in relating seminal zinc levels with semen parameters, but so far the results are inconclusive. Chia *et al*., reported a significant relationship of seminal plasma zinc with sperm density, motility and viability.[2] Similar studies have shown the relationships with volume,[15] pH,[16] concentration[17] and morphology.[18] Lack of relationships is also evident in some other studies.[3,5,19] Hence, there is no consensus on determination of the seminal plasma zinc levels in relation to semen quality or prostate function.

The objective of the present study was to determine the seminal plasma zinc levels in a subfertile population and to find the relationship between zinc and semen parameters using two markers; zinc concentration (Zn-C) and total zinc (Zn-T) in the ejaculate.

## SUBJECTS AND METHODS

### Subjects

Men who were referred for semen analysis at the university-based subfertility clinic were recruited for the study after obtaining informed consent. 330 males were interviewed and 152 were selected. Males with a history of STI, testicular injury, mumps-related orchitis, diabetes mellitus, small testicles (<10mL), varicocele, heavy or regular use of alcohol or smoking, reproductive endocrine abnormalities and fever within the past 3 months were excluded. Ethical clearance for the study was obtained from the ethics committee of the institute.

### Semen samples

Men were instructed to collect semen samples by masturbation after 3 days of sexual abstinence. Samples were allowed to liquify at 37°C for 30 mins and analyzed for descriptive semen parameters within 1 h of collection. Analysis was performed according to the WHO guidelines of 1999.[20] Immediately after assessing the volume, a portion of the sample was centrifuged at 5000g for 10 mins and the supernatant was stored at -20°C for determining the zinc levels. A second sample was obtained from pathozoospermics within 2 weeks of production of the first sample and was analyzed. Of the two samples, a sample with better Functional Motile Sperm Count (FMSC) was included in the study. FMSC was calculated by subtracting the total number of immotile sperm from the total sperm count.

### Determination of zinc

All glassware and plasticware were rinsed with 10% nitric acid overnight and thoroughly washed with deionized distilled water before use. Zn-C in seminal plasma was determined by flame atomic absorption spectrometry (GBC 932 AAS, JOEL Electronics, Inc., Tokyo, Japan). One milliliter of seminal plasma was digested overnight in 2 ml of concentrated nitric acid and 2 mL of perchloric acid. The supernatant was diluted 150-times with deionized water. Samples were run in the range of 0.5–1.5 ppm. Zn-C obtained was multiplied by ejaculate volume to calculate Zn-T. Intra and interassay coefficient of variation were 4.0 and 6.5%, respectively. WHO guidelines consider a Zn-T of <2.4 μmol (153 μg) in the human ejaculate as abnormal, and a cut-off value of <58.5 μg/mL for Zn-C was used in this study.

### Statistical analysis

Data analysis was performed using SPSS 10.0 for windows computer program. Mann Whitney U test and Chi-square test were used to compare the zinc levels between different seminal groups when appropriate. Correlations between numeric data were measured with Pearson’s correlation of coefficient. Statistical significance level was considered at *P*<0.05.

## RESULTS

Mean (SD) age of the study population was 33.43 years (5.1) and duration of subfertility was 39.09 months (33.62). The results of the basic semen analysis are summarized in Table 1.

**Table 1:** Characteristics of semen samples of the study population (*n* = 152)

Parameter	Mean (SD)	Number	%
Volume (mL)	2.73 (1.5)		
pH	7.85 (0.27)		
Concentration (mn/mL)	54.87 (49.64)		
Motility (%)	50.34 (18.48)		
Viability (%)	70.94 (16.02)		
Morphology (%)	39.51 (13.19)		
FMSC (mn)	31.41 (39.14)		
Normozoospermia		61	40.0
Pathozoospermia		91	60.0

FMSC, functional motile sperm count; mn/mL, millions per milliliter

The mean (SD) Zn-C and mean Zn-T in the ejaculate were 121.87 μg/mL (69.13) and 317.98 μg (227.92), respectively. Low zinc levels were found in 23 (15%) samples when Zn-C was used as the marker while the number of abnormal samples increased to 32 (21%) when Zn-T was used as the marker. The number of pathozoospermic samples was high in the low-zinc groups compared with the normal-zinc groups, irrespective of the marker used; 15 vs. 8 (*P* > 0.05) when Zn-C was used as the marker and 28 vs. 4 (*P* < 0.001) when Zn-T was used as the marker.

Seminal plasma Zn-C between the normal and the subnormal semen parameters [Table 2] showed that the mean Zn-C is significantly high in the low-motile group (asthenozoospermics) compared with the group with normal motility; 138.11 μg/mL (83.92) vs. 110.69 11 μg/mL (54.59) (*P* < 0.05). In contrast, Zn-C was not significantly different between the normal and the subnormal groups in relation to volume, pH, count, viability and morphology.

**Table 2:** Seminal plasma zinc levels between normal and subnormal semen parameters, mean (SD), *n* = 152

Parameter	Number	Zn conc in semen (μg/mL)	Total Zn per ejaculate (μg)	
pH	Normal (*n* = 150)	122.36 (69.39)	320.71 (228.11)
	Abnormal (*n* = 02)	85.20 (37.97)	113.21 (77.58)
Viscosity	Normal (*n* = 128)	126.16 (72.18)	336.34 (236.33)
	Abnormal (*n* = 24)	98.98 (44.20)	220.06(144.09)*
Count (mn/mL)#	Normal (106)	122.58 (69.26)	321.06 (233.87)
	Abnormal (37)	120.24 (69.55)	306.12 (219.67)
Motility (%)#	Normal (*n* = 90)	110.69 (54.50)	294.28 (195.88)
	Abnormal (*n* = 53)	138.11 (83.92)*	356.10 (275.40)
Viability (%)#	Normal (*n* = 100)	118.08 (66.07)	305.15 (221.19)*
	Abnormal (*n* = 43)	144.24 (83.26)	437.67 (283.88)
Morphology (%)#	Normal (*n* = 112)	116.58 (65.75)	307.60 (227.84)	
	Abnormal (*n* = 31)	113.67 (76.76)	351.86 (236.38)
FMSC (mn)#	Normal (*n* = 100)	119.83 (67.99)	325.82 (229.24)
	Abnormal (*n* = 43)	123.21 (66.98)	284.71 (231.94)
SFA category#	Normozoospermic (*n* = 55)	122.58 (69.26)	321.06 (233.87)
	Pathozoospermic (*n* = 88)	114.71 (62.96)	306.12 (219.67)
	Azoospermic (*n* = 9)	142.98 (92.98)	330.50 (210.99)

**P<0.01

* *P*< 0.05 compared with normal; mn/mL, millions per milliliter; FMSC, functional motile sperm count; SFA, seminal fluid analysis;

# Azoospermics were excluded

Zn-T was significantly low in samples with hyperviscosity compared with samples with normal viscosity; 220.06 μg (144.09) vs. 336.34 μg (236.33) (*P* < 0.05). Conversely, a significantly high Zn-T value was found in the low-viability group compared with the normal-viability group; 437.67 μg (283.88) vs. 305.15 μg (221.19) (*P* < 0.05) [Table 3].

**Table 3:** Pearson’s coefficient of correlations (*r*-values) for relationship between zinc levels and semen parameters (*n* = 152)

Parameter	Zn-C (μg/mL) r-value	Zn-T (μg) r-value
pH	−0.193*	−0.280**
Concentration (mn/mL) #	0.012	−1.000
Total count (mn) #	−0.078	0.211*
Motility (%)#	−0.122	−1.000	
Viability (%)#	−0.063	−0.087
Morphology (%)#	−0.113	−0.084

* *P*<0.05

** *P*<0.01; mn/mL, millions per milliliter;

# Azoospermics were excluded

A weak negative correlation (r = ∓0.193, *P* < 0.05) was found between Zn-C and semen pH. There were no correlations between Zn-C and any other semen parameters.

The correlation between Zn-T and semen pH was also inverse (r = -0.280, *P* < 0.01). On the other hand, a positive weak correlation was found between Zn-T and sperm count (r = 0.211, *P* < 0.05).

## DISCUSSION

The mean seminal plasma Zn-C in our study population (121 μg/mL) is comparable with some similar studies elsewhere in the world, e.g. Lewis Jones *et al*., (118 μg/mL),[3] Wong *et al*., (97.5 μg /mL)[21] and Kruse *et al*., (123 μg /mL).[7] However, the reported zinc levels in a few studies are considerably higher than that of our study; Omu *et al*., (170.6 μg/mL),[7] Umeyama *et al*., (171.1 μg/mL),[5] Omu *et al*., (171.1 μg/mL),[22] Chia *et al*., (183.6 μg/mL),[2] while lower levels were reported by Wong *et al*., (91.1 μg/mL),[16] and Tikkiwal *et al*., (78.9 μg/mL).[4]

Our results show that pathozoospermia is associated with low-seminal plasma zinc levels. Several factors are associated with seminal zinc deficiency. Inflammatory conditions considerably influence the secretory function of the prostate.[7] This may result in an impaired turnover and decreased secretion of zinc. Accumulation of toxic heavy metals in the testicular tissues may reduce the zinc levels in semen.[12,23] On the other hand, a low Zn/Cd ratio decreases the immune response of the semen or testis.[7] Many investigators have demonstrated that chronic prostatitis is associated with a drop in the zinc content in prostatic fluid.[16] Frequent ejaculation is another possible factor that can reduce the seminal plasma zinc levels. Seminal plasma zinc declines with zinc depletion. Severe zinc depletion causes a 50% decrease in the amount of zinc per ejaculate.[24] The low-zinc content of semen may affect the semen quality in different ways. Some mechanisms are reduced antioxidant capacity[11] and counteracting the effects of other heavy metals.[12]

We observed significantly different zinc levels between the normal and the subnormal groups in relation to viscosity, motility and viability. However, most of those were found with Zn-T and not with Zn-C. The only variation found with Zn-C was significantly high zinc levels in the asthenozoospermic group. This is consistent with the results of Fujiuchi *et al*.,[25] Mendoza *et al*.,[26] and Carpino *et al*.[8] This could be a reflection of elevated free zinc fraction due to incomplete post-ejaculatory redistribution of the zinc from the prostatic secretions (free and zinc-citrate complex) to higher affinity vesicular ligands. Zinc bound to high-molecular weight proteins (HMW-Zn%) is decreased in asthenozoospermic subjects, resulting in an increase in the free zinc in the seminal plasma. Impairment of motility in these samples may be due to interference by zinc ions on plasma membrane permeability and subsequent uptake by spermatozoa and interference in metabolic processes in sperm.[17] Using the results obtained by computer-assessed semen analysis (CASA), Sorensen *et al*., suggested that high seminal Zn-C has only a suppressing effect on progressive motility of spermatozoa (quality of movement) but not on percentage of motile spermatozoa (quantity of movement)[27] However, we observed comparable levels of Zn-T levels between the two above groups. Validity of measuring total seminal zinc (free and bound to vesicular HMW proteins) is questioned by Carpino *et al*.[8] They propose that HMW-Zn% has a better discriminating value among males with normal or low sperm motility.

Viscosity and viability are affected by variations of Zn-T. Lower Zn-T levels in hyperviscous samples are indicative of low-prostatic secretion, which contains prostate-specific antigen. On the other hand, zinc levels were high in the abnormal viability group, indicating the toxic effect of high-zinc levels on sperm vitality.

Available data on the correlation of zinc with semen parameters is also inconclusive. Positive correlations between seminal Zn-C have been reported with sperm concentration and motility,[25] with sperm density, motility and viability,[2] with sperm count[16] and duration of abstinence,[28] with sperm count of males with normal count but not in oligospermic males.[29] Some other authors have shown that there is no correlation between the total amount of zinc or Zn-C and semen characteristics.[7,17,27]

In this study, Zn-C showed a weak inverse correlation with pH. There was a weak positive correlation between Zn-T and count; in contrast, there was an inverse correlation with pH. Seminal zinc levels seem to reflect the spermatogenic activity of the testis, as shown by a positive correlation between sperm count and Zn-T. The number of germinal cells and sperms are reported to reduce in the testes of zinc-deficient rats. This is partly due to the low activity of angiotensin-converting enzyme, a zinc metallo enzyme that is important in sexual development (maturation of testis) and fertility in males of many species.[30]

Increased levels of zinc in seminal plasma cause an increase in the intraspermatozoal zinc levels. As the correlation between zinc and pH is negative, we propose that intracellular pH may reduce with an increase in the seminal plasma zinc. The pH depression causes a reduction of respiration, motility and acrosome reaction (AR)[31] of the spermatozoa. This may have some advantage for the spermatozoa to stay in a relatively inactive state until activated in the female reproductive tract as diluted in the vaginal fluid. Furthermore, the fusion of prostasomes and spermatozoa is pH and protein dependent. Usually, the fusion has been investigated with the pH levels below eight and fusion increases with lowering pH. These prostasomes contain many small ions (Ca^2+^, Zn^2+^, GDP, ADP and ATP) and a number of enzymes. The fusion is helpful to transfer a number of molecules into spermatozoa, modifying the sperm membrane lipoprotein pattern and regulating some important functions (motility, AR) of the spermatozoa.[32,33]

Our results clearly indicated that there is a relationship between zinc and semen quality. However, further studies are needed in our population to observe whether supplementation improves the semen quality in males with subnormal zinc levels or subnormal semen parameters. Such studies will be useful in deciding the necessity of assessing seminal plasma zinc levels in the evaluation of male subfertility.

## CONCLUSION

According to the results, the andrological variables sensitive to seminal plasma zinc variations are motility, count, viability, pH and viscosity. High Zn-C showed a negative effect on motility and elevated Zn-T negatively affected the viability. There was a positive relationship between Zn-T and total sperm count. Seminal plasma Zn-T appears to be the better marker for assessing the relationship between zinc and semen quality compared with Zn-C.
